# The Role of Ethnic Disparities in the Outcomes of Bariatric Surgery: A Systematic Review and Meta-Analysis

**DOI:** 10.7759/cureus.24743

**Published:** 2022-05-05

**Authors:** Mohamed Aysar Khattab, Abdelrahman Tarek Abdelnaby Mohammed, Abdulrahman Zaid M Alqahtani, Ebtehal Zaid M Alqahtani, Manar Mohammed A Alslim, Nawaf Essa A Alharbi, Rana Mohammed A Alslim, Zobaida Saleh, Mohammed Ali Qassim Atia, Albaraa Jubran Shanaq, Abdelwahab Saleh

**Affiliations:** 1 Department of Anesthesiology and Critical Care, Al-Azhar University, Cairo, EGY; 2 Department of Pharmacy, Faculty of Pharmacy, Jazan University, Jazan, SAU; 3 Department of Vascular Surgery, Faculty of Medicine, Jazan University, Jazan, SAU; 4 Department of Pharmacy, Faculty of Pharmacy, Princess Nourah Bint Abdulrahman University, Riyadh, SAU; 5 Department of Pharmacy, Faculty of Pharmacy, Shaqra University, Shaqra, SAU; 6 Department of Pharmacology and Toxicology, Faculty of Pharmacy, Mohammad Al-Mana College of Health Sciences, Al-Khobar, SAU

**Keywords:** metabolic outcomes, gastric bypass, sleeve gastrectomy, bariatric surgery, obesity, hispanics, race, racial disparities, ethnicity

## Abstract

Bariatric surgery is increasingly performed over the past decade for the treatment of morbid obesity. It has beneficial effects on weight reduction, along with diabetes remission. Conflicting results have been reported to evaluate the effect of ethics differences on the outcomes of bariatric surgery. We conducted this meta-analysis to outline the effects of ethnic differences on the outcomes of bariatric surgery, including weight reduction, biochemical variables, diabetes, and hypertension remission. A comprehensive literature search was conducted, using PubMed, Web of Science (ISI), Google Scholar, Popline, Global Health Library (GHL), Virtual Health Library (VHL) including Cochrane database, New York Academy of Medicine (NYAM), and System for Information on Grey Literature in Europe (SIGLE) for studies reporting body mass index (BMI), percentage of excess weight loss (%EWL), waist circumference, hypertension, lipid profile, and diabetes variables. We used the National Heart, Lung, and Blood Institute (NHLBI) tool (Bethesda, MD: NHLBI, National Institutes of Health {NIH}) for quality assessment. Comprehensive Meta-Analysis version 2 software (Englewood, NJ: Biostat, Inc.) was applied to perform the meta-analysis of the variables of interest. We included 23 studies of 71,679 subjects, who underwent bariatric surgery. The majority of the included cases were Whites 55,030 (77%), while 705 (1%) were Asians. The percentages of Blacks, African Americans, Hispanics, and Non-Hispanics were 9.3%, 1.3%, 10.4%, and 1%, respectively. BMI showed no significant difference between Whites vs African American and Hispanic vs Non-Hispanic groups (MD: 0.858; 95% CI: 3.408-1.691; p = 0.509 and MD: 0.455; 95% CI: 2.444-1.554; p = 0.663, respectively). The same result was reported for %EWL, comparing Whites vs African Americans. Lipid biochemical variables, diabetes remission, and hypertension control were significantly more seen among the Asian population. In conclusion, we reported a significant ethnic diversity and reduction in waist circumference, hyperlipidemia, and the associated morbidity one year after bariatric surgery in the Asian population. Further, high-quality prospective studies should focus on the social and psychological ethnic differences associated with obesity.

## Introduction and background

Obesity and its associated morbidity represented a major health and economic burden in both developed and developing countries [1]. Obesity control can be achieved through multiple modalities, including lifestyle modification, dietary management, pharmacological intervention, and bariatric surgery. Indeed, bariatric surgery remains the sole long-standing, most effective treatment for morbid obesity [2]. Importantly, bariatric surgery has multiple beneficial effects, not only on weight reduction but also on remission of type 2 diabetes mellitus (DM-2) [3]. Indeed, bariatric surgery is increasingly performed over the past decade, which can be attributable to the latest advancement in procedure techniques. In addition, the morbidity and mortality associated with bariatric surgery have declined in the past few years, contributing to the popularity of the procedure [4]. It has been suggested that the outcomes of bariatric surgery differ inter-individually. Moreover, scarcity of data reported that bariatric surgery may be influenced by ethnic diversity. The objective of this study was to systematically review and meta-analyze the data pooled on the ethnic differences in the metabolic outcomes, diabetes remission, and weight reduction after bariatric surgery.

## Review

Methods

This meta-analysis was conducted in accordance with the Preferred Reporting Items for Systematic Reviews and Meta-Analysis (PRISMA) statement [5]. Conduction of the study did not require ethical approval according to our institution's policy.

Eligibility Criteria

We included all the studies that discuss the effects of ethnicity on the outcomes of bariatric surgery. No restrictions were considered regarding the population age, sex, language, race, place, and publication date. We excluded editorial comments, thesis, reviews, book chapters, news, and only-abstract articles. Articles with no relevant data or data that can not be extracted were also excluded.

Search Strategy and Study Selection

A comprehensive literature search was conducted using PubMed, Web of Science (ISI), Google Scholar, Popline, Global Health Library (GHL), and Virtual Health Library (VHL), including Cochrane database, New York Academy of Medicine (NYAM), and System for Information on Grey Literature in Europe (SIGLE), for the relevant articles. We used the following search terms - "Ethnicity OR Race OR 'ethnic minority' OR 'racial disparities' OR 'ethnic variation' OR Whites OR Blacks OR Asians OR Hispanics" AND "bypass surgery" OR "gastric bypass" OR "bariatric surgery" OR "sleeve gastrectomy" OR Roux-en-Y. The search string was modified in accordance with each database. Upon retrieval of the search results, we used EndNote X7.4 software (London, UK: Clarivate) to remove the duplicates. Title and abstract screening were performed by two independent reviewers, then followed by full-text screening to finally include the relevant articles according to our inclusion and exclusion criteria. Then, we manually searched the references of the included articles to add further relevant studies. Any discrepancy was resolved by a third senior reviewer.

Data Extraction

Two independent reviewers extracted the relevant data from the finally included studies. Any discrepancy was resolved by a senior third reviewer. The extraction was performed upon a standardized extraction form. It was formulated through pilot extraction of three of the included studies to show the variables of interest. We extracted variables related to the demographic characteristics of the patients included, such as the study comparison, age, sex, ethnicity, type of surgery, and BMI. We also extracted variables showing the effects of ethnicity on the outcomes of bariatric surgery, including BMI, %EWL, waist circumference, hypertension, lipid profile, and diabetes variables. Graphically presented data was extracted using PlotDigitizer software (CA, USA: SourceForge) (http://plotdigitizer.sourceforge.net/).

Risk of Bias Assessment

Two independent reviewers assessed the risk of bias in the included articles using the National Heart, Lung, and Blood Institute (NHLBI) tool (Bethesda, MD: NHLBI, National Institutes of Health {NIH}) for quality assessment. Any discrepancy was resolved by a third senior reviewer. Fourteen well-organized questions were proposed to assess the study population, sample size, exposure, outcome, follow-up, and confounding variables.

Statistical Analysis

We used Comprehensive Meta-Analysis software (version 2) to perform the meta-analysis. The random-effect model was manipulated with significant heterogeneity, while the fixed effect model was used with the absence of significant heterogeneity. Statistical heterogeneity was assessed using χ2 test and I^2^ statistics. Significant heterogeneity was considered when χ2 test has a p-value < 0.1 or I^2^ test value > 50%. Mean difference (MD) and 95% confidence interval (95% CI) were used to present continuous data, otherwise, standardized mean difference (SMD) was suitable.

Results

Search Results and Characteristics of Included Studies

**Figure 1:**
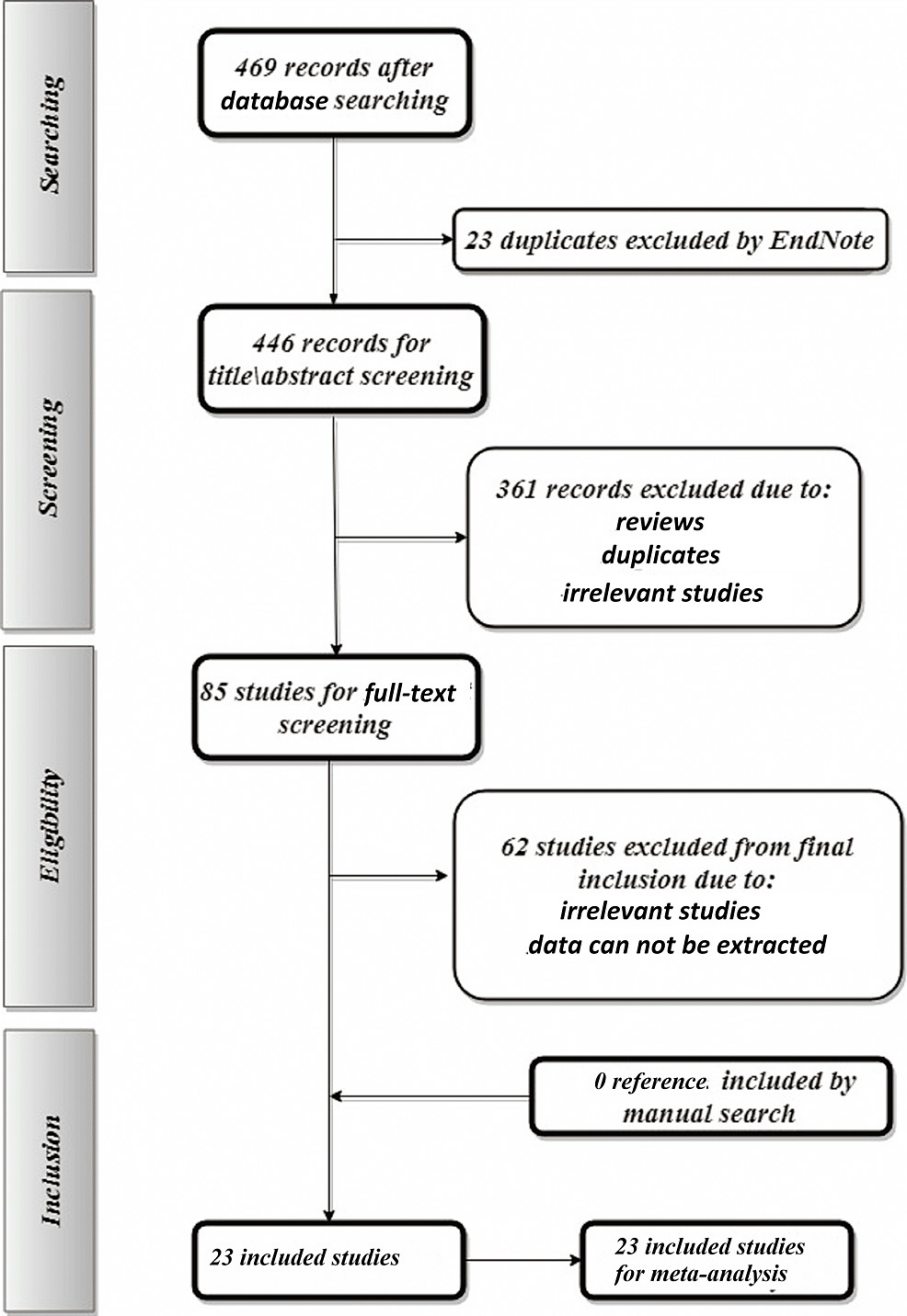
Flow chart illustrating the sequence procedure of the included articles.

We identified 469 reports from the initial search of different databases, of which, 23 reports were excluded by EndNote software as duplicates. Title and abstract screening of the remaining 446 reports revealed the exclusion of 361 reports for different reasons. We eventually included 23 reports in the meta-analysis after the exclusion of 62 reports by full-text screening (Figure 1).

We included 71,679 subjects, who underwent bariatric surgery, to meta-analyze the effects of ethnicity on the outcomes of the bariatric surgery. The majority of the included cases were Whites 55,030 (77%), while 705 (1%) were Asians. The percentages of Blacks, African Americans, Hispanics, and Non-Hispanics were 9.3%, 1.3%, 10.4%, and 1%, respectively. Table 1 represents the characteristics and demographic data of the included studies.

**Table 1:** Demographic characteristics of the included studies. %EWL: percentage of excess weight loss

Author, year		Study design	Type of surgery (%)	Ethnicity	Sample N (%)	Age mean (SD)	% of female	BMI (kg/m^2^) mean (SD)	%EWL mean (SD)
Admiraal et al., 2013 [6]		Retrospective database review	Roux-en-Y gastric bypass (100)	Black	48 (30.6)	41.8 (10.1)	NA	45.2 (6.2)	55.7 (17.8)
				Asian	43 (27.4)	43.0 (9.3)	NA	44.4 (6.9)	58.2 (18.3)
				White	66 (42)	38.3 (9.1)	NA	46.1 (5.2)	58.3 (17.7)
Anderson et al., 2007 [7]		Retrospective database review	Roux-en-Y gastric bypass (100)	African American	24	41 (10)	79	55 (10)	72.6 (10.0)
				White	61	43 (10)	75	53 (11)	61.7 (8.0)
Araia et al., 2014 [8]		Retrospective database review	Sleeve gastrectomy (34)	African American	597 (100)	42.6 (8.0)	73	54.1 (7.3)	NA
			Gastric banding (21.8)	African American	597 (100)	42.6 (8.0)	73	54.1 (7.3)	NA
			Roux-en-Y gastric Bypass (44.2)	African American	597 (100)	42.6 (8.0)	73	54.1 (7.3)	NA
Bayham et al., 2012 [9]		Retrospective chart review	Roux-en-Y gastric bypass (48)	White	325 (77)	40.2 (10.9)	100	46.5 (7.3)	36.1 (7.7)
			Sleeve gastrectomy (52)	Black	95 (23)	37.9 (9.5)	100	47.2 (7.5)	34.6 (8.6)
Di et al., 2016 [10]		Retrospective database review	Roux-en-Y gastric bypass (100)	Asian	66 (100)	50.4 (11.4)	57.6	28.2 (1.2)	NA
Guajardo-Salinas et al., 2008 [11]		Retrospective database review	Roux-en-Y gastric bypass (100)	White	26 (35)	54 (NA)	84	48 (NA)	77.35 (NA)
				Hispanic	49 (65)	43 (NA)	84	50 (NA)	83 (NA)
Gullick et al., 2015 [12]		Retrospective database review	Roux-en-Y gastric bypass (100)	African American	170 (26)	41.6 (9.6)	90	51.7 (7.4)	58.3 (13.2)
				White	493 (74)	42.2 (9.5)	73.4	48.5 (7)	70.0 (14.1)
Khorgami et al., 2015 [13]		Retrospective chart review	Roux-en-Y gastric bypass (88.7)	Hispanic	1561 (48)	41.4 (13.2)	74.6	46.2 (7.6)	66.0 (20.3)
			Gastric banding (11.3)	Non-Hispanic	660 (20)	40.6 (11.0)	87.9	48.5 (9.2)	54.1 (21.3)
Malapan et al., 2014 [14]		Prospective cohort	Roux-en-Y gastric bypass (100)	Asian	29 (100)	53 (NA)	55.2	24.4 (1.8)	NA
Mazidi et al., 2017 [15]		Prospective cohort	Roux-en-Y gastric bypass (100)	Asian	152 (100)	42.7 (8.7)	64.1	30.31 (5.38)	NA
Mazidi et al., 2017 [16]		Prospective cohort	Roux-en-Y gastric bypass (100)	Asian	209 (100)	NA	61.5	29.98 (5.45)	NA
Mui et al., 2008 [17]		Prospective cohort	Sleeve gastrectomy (100)	Asian	70 (100)	34.7 (8.8)	49	40.7 (7.8)	63.5 (29.4)
Ng et al., 2015 [18]		Retrospective database review	Roux-en-Y gastric bypass (25.7)	African American	302 (17.9)	42.7 (10.1)	92.1	46.6 (7.1)	NA
				White	1145 (68)	47.0 (11.1)	76.8	46.1 (7.1)	NA
			Gastric banding (74.3)	Hispanic	237 (14.1)	37.9 (8.6)	86.9	46.0 (6.2)	NA
Omotosho et al., 2016 [19]		Nested case-control study	Roux-en-Y gastric bypass (100)	African American	78 (27.7)	40.3 (9.1)	100	50.6 (7.5)	50.2 (7.9)
				White	204 (72.3)	41.1 (8.9)	100	50.2 (7.1)	60.5 (4.3)
Parikh et al., 2006 [20]		Prospective cohort	Gastric banding (100)	African American	58 (47.2)	37 (19)	NA	47 (7)	39 (19)
				White	65 (52.8)	37 (19)	NA	47 (7)	49 (18)
Stanford et al., 2015 [21]		Prospective cohort	Gastric bypass surgery	White	367 (71.7)	45.6 (NA)	74	46.2 (NA)	NA
			Gastric banding	African American	87 (17)	41.9 (NA)	89	48.7 (NA)	NA
			Sleeve gastrectomy	Hispanic	58 (11.3)	35.8 (NA)	76	47.6 (NA)	NA
Sudan et al., 2014 [22]		Retrospective database review	Roux-en-Y gastric bypass surgery (100)	Black	6,286 (10)	42.7 (10.57)	85	35.0 (7.51)	NA
				Hispanic	4,723 (7.5)	41.0 (11.10)	78	32.6 (7.03)	NA
				White	51,953 (82.5)	46.4 (11.62)	77	31.6 (6.73)	NA
Wee et al., 2017 [23]	Retrospective cohort		Roux-en-Y gastric bypass (54)	White	325 (71.1)	46.3 (NA)	74	46.3 (NA)	34.48 (8.65)
				African American	80 (17.5)	42.6 (NA)	88	48.5 (NA)	29.77 (7.38)
			Gastric banding (46)	Hispanic	52 (11.4)	35.7 (NA)	75	47.9 (NA)	31.79 (10.18)
Yin et al., 2014 [24]		Retrospective cohort	Roux-en-Y gastric bypass (100)	Asian	68 (100)	49.2 (11.0)	63.2	31.0 (7.3)	NA
Yu et al., 2015 [25]		Retrospective cohort	Roux-en-Y gastric bypass (100)	Asian	68 (100)	47.8 (11.7)	57.4	31.5 (3.6)	NA
De La Cruz-Muñoz et al., 2013 [26]		Retrospective database review	Roux-en-Y gastric bypass (92)	Hispanic	57 (80)	18.3 (1.04)	80	45.8 (5.3)	28.6 (16.9)
			Gastric banding (8)	Non-Hispanic	14 (20)	18.3 (1.04)	78.6	47.3 (5.6)	31.0 (39.6)
De La Cruz-Muñoz et al., 2010 [27]		Retrospective database review	Roux-en-Y gastric bypass (91)	Hispanic	60 (76.9)	NA	77	47.71 (1.51)	NA
			Gastric banding (7.7)	Non-Hispanic	15 (19.2)	NA	84	49.24 (2.35)	NA
De La Cruz-Muñoz et al., 2013 [28]		Retrospective database review	Roux-en-Y gastric bypass (91)	Hispanic	633 (100)	41.1 (12.5)	76	46.4 (7.5)	NA

Risk of Bias Assessment

Risk of bias assessment revealed that the quality of the included studies ranged from fair to poor. The most reported items of high risk of bias were the inadequate blinding of the outcome assessors, exposing the results to detection bias. Randomization was not ensured and most of the studies were not sufficiently sized to answer the pre-specified question. In addition, the key potential confounding variables were not statistically considered in most of the studies.

Outcomes

**Figure 2:**
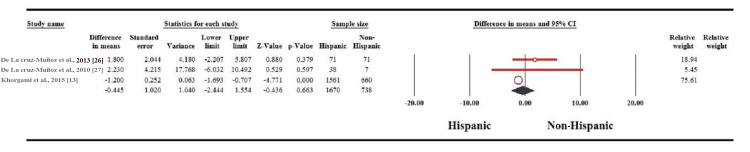
Forest plot of the changes in BMI between Hispanics and non-Hispanics.

**Figure 3:**
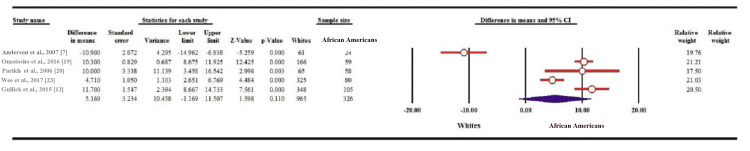
Forest plot of the change in %EWL. %EWL: percentage of excess weight loss

Variables eligible for meta-analysis were BMI, %EWL, waiste circumference, total cholesterol, triglycerides, low-density lipoprotein (LDL), high-density lipoprotein (HDL), fasting blood glucose, HbA1c, systolic and diastolic blood pressure. Regarding BMI, there was no statistically significant difference between Whites vs African American and Hispanic vs Non-Hispanic groups (MD: 0.858; 95% CI: 3.408-1.691; p = 0.509 and MD: 0.455; 95% CI: 2.444-1.554; p = 0.663, respectively) (Figure 2). Percentage of EWL did not show a significant difference between Whites and African American groups (MD: 5.169; 95% CI: 1.169-11.507; p = 0.110) (Figure 3).

**Figure 4:**
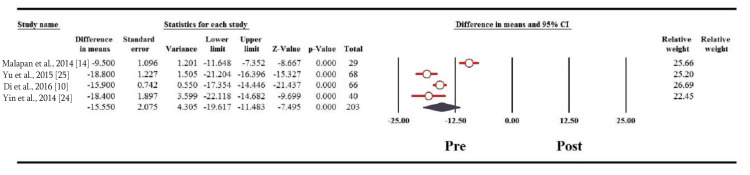
Forest plot of the changes in waist circumference.

**Figure 5:**
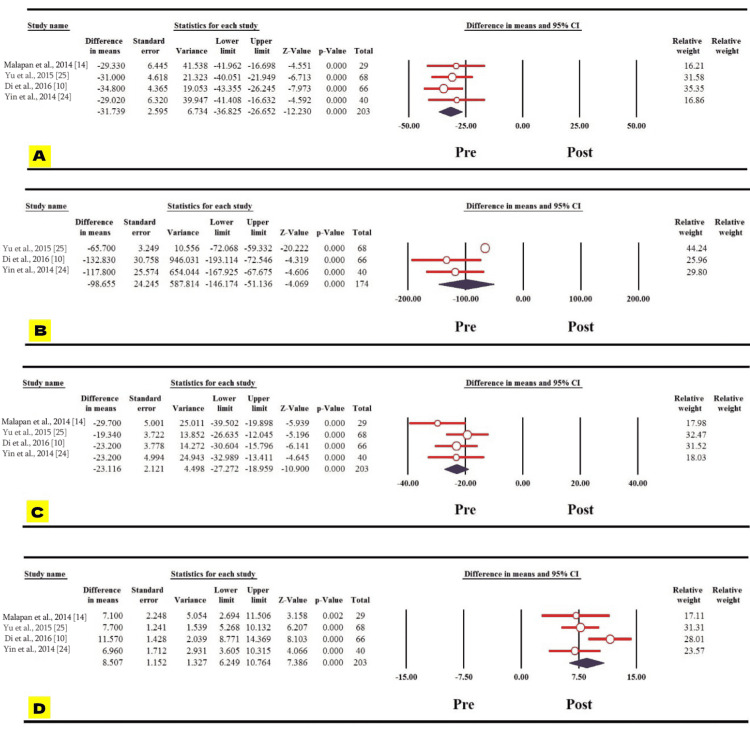
Forest plot of the changes in the lipid profile before and one year after bariatric surgery. Forest plots of (A) total cholesterol, (B) triglycerides, (C) LDL, and (D) HDL. LDL: low-density lipoprotein; HDL: high-density lipoprotein

Interestingly, waist circumference showed significant decrease one year following bariatric surgery among the Asian population (MD: 15.550; 95% CI: 19.617 to -11.483; p = 0.000) (Figure 4). Other biochemical variables, including total cholesterol, triglycerides, and LDL decreased significantly one year after the surgery among the Asian population, as shown from the pooled mean differences (MDs) of the three variables (MD: 31.739, 95% CI: 36.825 to -26.652, p = 0.000; MD: 98.655, 95% CI: 146.174 to -51.136, p = 0.000; MD: 23.116, 95% CI: 27.272 to -18.959, p = 0.000, respectively). Conversely, there was a significant increase of HDL one year post-operatively among the Asian population (MD: 8.507; 95% CI: 6.249-10.764; p = 0.000) (Figures 5A-5D).

**Figure 6:**
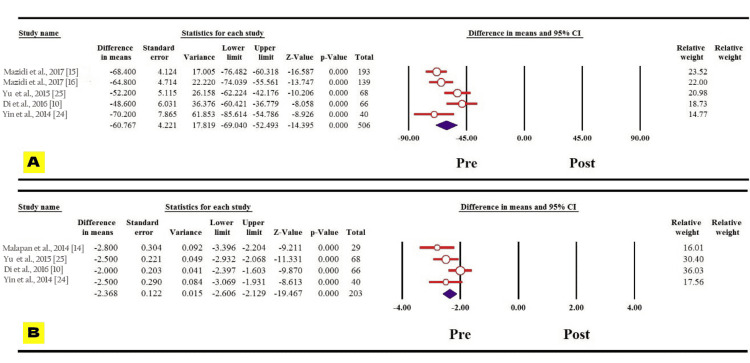
Forest plot of the changes in (A) fasting blood glucose and (B) HbA1c.

**Figure 7:**
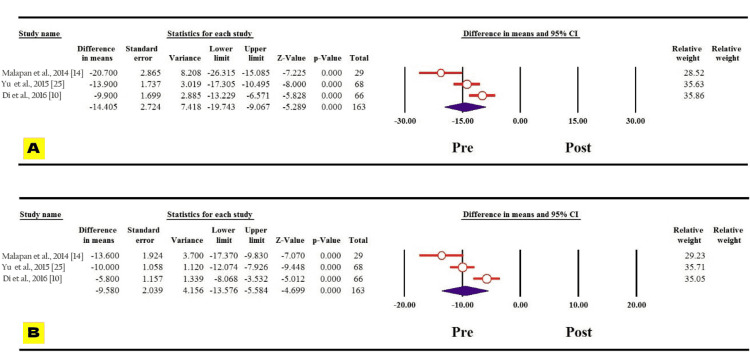
Forest plot of the changes in (A) systolic blood pressure and (B) diastolic blood pressure.

Diabetes variables like fasting blood glucose and HbA1c decreased significantly one year following bariatric surgery among the Asian population evidenced by the reported MDs (MD: 60.767, 95% CI: 69.040 to -52.493, p = 0.000; MD: 2.368, 95% CI: 2.606 to -2.129, p = 0.000, respectively) (Figures 6A, 6B). Regarding blood pressure, the systolic and diastolic blood pressures also showed statistically significant decrease one year following the surgery among the Asian population (MD: 14.405, 95% CI: 19.743 to -9.067, p = 0.000; MD: 9.580, 95% CI: 13.576 to -5.584, p = 0.000, respectively) (Figures 7A, 7B). 

Discussion

This study aimed to discuss the association between ethnic variations and the metabolic outcomes of bariatric surgery. We reported no statistically significant difference between Whites vs African American and Hispanic vs Non-Hispanic groups regarding BMI and %EWL. In addition, the Asian population showed a significant decrease in the waist circumference one year following bariatric surgery regardless of the type of the procedure. Indeed, previous studies reported conflicting results regarding the association of race and weight loss indices. In a retrospective cohort study conducted by Wood et al., they questioned 14,000 patients undergoing bariatric surgery for the ethnic differences and the relation to weight loss one year after the surgery [29]. They demonstrated that weight loss was more dominant in the Whites than the African American patients. Nevertheless, the difference noted was modest. Moreover, another retrospective study conducted by Sudan et al. investigated more than 100,000 patients undergoing Roux-en-Y gastric bypass [22]. Similarly, they found that the mean percent change in BMI was less demonstrated in the African Americans as compared to the White patients. In addition, lower weight loss was frequently reported in several studies after bariatric surgery [30,31]. Indeed, our results supported that weight loss after bariatric surgery is independent of the surgeon and the site of the operation rather than the ethnic variations. The reason behind this difference is not clearly elucidated. Nevertheless, biological, behavioral, and socioeconomic status may represent contributing factors to this difference [29]. It has been reported that the daily resting energy expenditure is lower among African Americans compared to their counterparts [32]. The difference in weight loss between Whites and African Americans after bariatric surgery can be postulated by the metabolic diversity between the two groups. However, the study conducted by Luke et al. inquired about the causal relationship between reduced weight loss and low resting energy expenditure in African Americans [33]. They found that either activity or resting energy expenditure is unlikely to have a role in weight gain in African Americans. They recommended that the clinical evaluation of the modifiable factors like energy intake and the level of physical activities remained the main relevant factors to weight loss or gain, rather than the biochemical metabolic nature of each ethnic group. Furthermore, the economic and sociodemographic factors may contribute to weight loss diversity after bariatric surgery [34]. The study conducted by Baker et al. investigated the availability and accessibility of multiple ethnicities to healthy food resources, and their relation to obesity [35]. They reported that African Americans, regardless of income, and poor White areas lacked adequate spatial distribution of healthy food resources, which may explain the different weight loss after bariatric surgery. In addition, social interference and behavioral challenges differ by ethnicity and may contribute to different response to bariatric surgery [36]. It has been reported that White patients expressed less satisfaction with obesity and higher social impairment compared to obese African Americans, which drives the Whites to lose more weight. In addition, another study assessed the psychological aspects and social interaction in a focus group of obese Black women. Black women reported less attractive feelings with losing weight [37]. Surprisingly, they were exposed to social pressure from their surroundings as a result of losing rather than gaining weight. Regarding the associated comorbidities, hypertension and diabetes variables like fasting blood glucose level and HbA1c decreased significantly one year after bariatric surgery. Supportingly, the retrospective study conducted by Lee et al. reported that within two years after bariatric surgery, there was significant remission in DM-2 in all the ethnic groups [38]. In the subsequent follow-up period, HbA1c steadily rise to the baseline values in the African American population. They supported the theory that insulin resistance, characteristic of DM-2, is preceded by hyper-insulin secretion [39]. Accordingly, this may explain the ethnic differences in DM remission after bariatric surgery. Conversely, the study conducted by Admiraal et al. reported no significant difference in remission of DM one year after bariatric surgery [6]. This conflicting result can be postulated by the lower studies included by Admiraal et al. as they included only three studies for assessment of DM remission, while the present study included five studies with higher sample size and more reliable results.

Limitations

Multiple confounding factors should be considered in further original articles, like baseline diabetes values, duration of diabetes, and anti-diabetic medications. Future studies should focus on the assessment of the psychological and behavioral aspects to precisely define the determinants of the ethnic differences after bariatric surgery.

## Conclusions

This meta-analysis confined the relationship between ethnic variations and the one-year outcomes of bariatric surgery. We reported significant ethnic diversity and reduction in waist circumference, hyperlipidemia and the associated morbidities one year after bariatric surgery. Further, high-quality prospective studies should focus on the social and psychological ethnic differences associated with obesity. Prolongation of the period of outcome assessment is encouraged to the ethnic variations and the long-term outcomes of bariatric surgery.
